# A short mental health literacy scale: validation and application in Brazil

**DOI:** 10.1186/s12889-025-24632-w

**Published:** 2025-10-08

**Authors:** Bernardo Oliveira Buta, Vilma V.D. Couto, Matheus Britto Froner, Benjamin Miranda Tabak

**Affiliations:** 1 School of Public Policy and Government, Getulio Vargas Foundation FGV/EPPG, SGAN 602 - Módulos A, B e C, Brasilia, DF 70830-020 Brazil; 2 Department of Psychology, Universidade Federal do Triângulo Mineiro, Av. Frei Paulino, n° 30 - Bairro Abadia, Uberaba, MG 38025-180 Brazil

**Keywords:** Mental health literacy, Mental health, Scale, Validation, Structural equation modeling

## Abstract

**Objective:**

To validate a version of the Mental Health Literacy Scale (MHLS) adapted to Brazilian Portuguese, and evaluate the level of mental health literacy in a sample of the Brazilian population.

**Methods:**

We applied the Brazilian Portuguese version of the MHLS to a sample of 472 adults. The questionnaires were self-reported by the participants in the presence of the researchers. We use nonparametric tests to compare literacy scores. Using confirmatory factor analysis, we tested the hypothesis of unidimensionality of the MHLS. The structure that best fit the version of the MHLS in Brazilian Portuguese was extracted through exploratory factor analysis.

**Results:**

The results showed that the model with a single-factor structure did not present a good fit. The Brazilian Portuguese adaptation of the MHLS displayed a four-factor structure, comprising 18 items. This version demonstrated satisfactory factor loadings and high levels of reliability. These characteristics suggest that the tool maintains internal consistency and reliability, and can be used to assess mental health literacy effectively. The average MHLS score of the sample was 123.01. The value of the score varies depending on gender, age, education, and the type of health service used.

**Conclusion:**

The Brazilian adaptation of the MHLS has been effective in gauging mental health literacy levels among Brazilians. Future efforts to enhance mental health literacy should focus on middle-aged people with lower education levels who utilize public services.

## Introduction

Mental health disorders are widespread on a global scale. Approximately 12.5% of the global population suffers from mental disorders, which are a leading cause of suicide, particularly among young people. Suicides represent more than 1% of all deaths [1]. The effects on families are devastating, and the impact on organizations is significant. The economic toll of mental disorders is substantial, often resulting in absenteeism and decreased work productivity [2].

The breadth of mental health literacy (MHL) contributes significantly to prevention, early intervention, self-help and support for people with mental disorders and those in their social circle [3, 4]. On the other hand, low levels of mental health literacy can affect the search for treatment and self-management of psychological problems. This can lead to delays or refusal by individuals with mental disorders to seek help and treatment [5–7].

Measuring MHL allows the identification of specific topics on public health that require strategic interventions. Targeted public policies can lead to greater efficiency by addressing identified needs directly. Ultimately, this approach can reduce the health system’s costs related to delayed treatments and hospitalizations. It is important to note that governments in developed Western countries have already undertaken efforts to measure and promote health literacy. This practice is crucial for evidence-based decision-making and the advancement of healthcare [8]. Adapting the MHLS for Brazil, which currently lacks instruments and policies in this area, could greatly enhance the development of public mental health policies.

MHL is a multidimensional and multilevel construct. It was introduced as the knowledge and beliefs about mental disorders that help their recognition, management and prevention. In this sense, it has several dimensions related to abilities, knowledge and attitudes [9]. Since then, several attempts to measure the level of mental health literacy have been made, some of which have expanded the construct to include dimensions related to behaviors, social stigma and access to mental health treatments [10–13].

The level of MHL varies according to the individual’s socioeconomic characteristics. Variations are observed according to gender[14–20], age[14–16, 19, 20], education [13, 14, 16–20], income [19, 20], and cultural context [18]. Furthermore, people who have a previous diagnosis of a mental disorder [11, 14, 17, 18, 20], or family members of these people [11, 14, 17, 18], and healthcare practitioners [11, 18] also tend to have higher levels of mental health literacy.

The Mental Health Literacy Scale (MHLS), proposed by O’Connor and Casey (2015), is relevant to the literature on the topic [21], has been validated in different cultural contexts [16, 17, 19, 22, 23]. Despite addressing several aspects of the MHL, its internal structure has only one dimension, which has already been questioned by other authors[16, 17, 22, 23], and can result in the loss of relevant information when the MHL is measured, making it difficult to identify which aspects of literacy individuals need greater support.

This study aimed to validate a version of the MHLS adapted to Brazilian Portuguese, and evaluate the mental health literacy of a sample of the Brazilian population. Furthermore, we test the internal structure of the MHLS and propose a shorter version of this instrument.

## Method

### Study design and ethical considerations

We applied the Brazilian Portuguese version of the MHLS to two samples. First, we performed a pilot application with 156 public servants [24]. The findings indicated the need for wording adjustments in eleven items, which were carried out based on the guidelines of Sadock et al. (2017), with the support of a doctor in psychology and specialist in mental health.

In addition to the pilot application, one other was carried out using the digital tool SurveyMonkey. The second application was performed in person on 472 ordinary people over 18 years old between September and October 2023. The data collection occurred at various locations with large flows of people within the Federal District to capture the heterogeneity of the population. The participants completed the questionnaires through self-reporting in the presence of the researchers, who could interact and answer any questions that arose. However, the researchers did not enter the information into the questionnaires themselves.

In addition to the MHLS, the questionnaire included items related to the sociodemographic characteristics and information concerning health services, medication use, and previously diagnosed health conditions. These covered gender, age, color/race, education level, employment status, income, housing conditions, use of health services, medication usage, and previously diagnosed health conditions.

Finally, it is important to state that the research protocols were reviewed for ethical compliance by the Fundação Getúlio Vargas’s Committee on Ethical Compliance in Research Involving Human Beings before data collection began, which approved the research.

### Participants

The sample consisted of 472 subjects, 43% men and 56.8% women. The median age was between 26 and 30 years. Regarding color/race, 41.9% were mixed race, 38.3% white, 18.2% black and 1.5% Asian or indigenous. Regarding educational level, 36.4% had completed higher education. 55.1% of the subjects were unemployed or informally employed, and 67.8% reported an income of up to three times the minimum wage (R$3,960). All participants returned to all items, so there were no missing data. The mean MHLS score for the sample is 123.01, ranging from 64 to 155, with a standard deviation of 13.87 (skewness: −0.685; kurtosis: 0.684). Table 1 summarizes the sociodemographic profile of the sample.

**Table 1:** Sociodemographic profile of the sample

Feature	Count	%	Median MHLS	Stat. Test (*p*-value)
**Gender**				
Male	203	43.0%	123	W = 32728 (< 0.001)
Female	268	56.8%	126	
Other	1	0.2%		
**Age**				
18-25	159	33.7%	129	KW = 39.015 (< 0.001)
26-30	81	17.2%	127	
31-35	60	12.7%	124.5	
36-40	38	8.1%	123.5	
41-45	35	7.4%	124	
46-50	44	9.3%	118.5	
50+	55	11.7%	129	
**Color/Race**				
Mixed race	198	41.9%	123	KW= 8.283 (0.08172)
White	181	38.3%	127	
Black	86	18.2%	126.5	
Asian	4	0.8%	127.5	
Indigenous	3	0.6%	122	
**Education**				
No formal education	2	0.4%	100	KW = 45.958 (< 0.001)
Incomplete Primary Education	17	3.6%	116	
Complete Primary Education	11	2.3%	113	
Incomplete Secondary Education	24	5.1%	114	
Complete Secondary Education	132	28.0%	123	
Incomplete Higher Education	114	24.2%	129	
Complete Higher Education	172	36.4%	126	
**Labor situation**				
Unemployed	135	28.6%	124	KW = 0.010 (0.9947)
Informally Employed	125	26.5%	125	
Formally Employed	212	44.9%	125	
**Income**				
Up to 1 minimum wage (R$ 1.320)	126	26.7%	127	KW = 7.801 (0.167)
From 1 to 3 minimum wages (R$1.320 a R$3.960)	194	41.1%	124	
From 3 to 6 minimum wages (R$3.960 a R$7.920)	86	18.2%	125.5	
From 6 to 9 minimum wages (R$7.920 a R$11.880)	49	10.4%	124	
From 10 to 20 minimum wages (R$13.200 a R$26.400)	14	3.0%	123.5	
More than 20 minimum wages (above R$26.400)	3	0.6%	121	
**Housing condition**				
Live alone	83	17.6%	123	W = 15098 (0.354)
Live with others	389	82.4%	125	
**Use of health services**				
Private health services	118	25.0%	126.5	D= 2.846 (0.002)
Public health services	186	39.4%	123	
Public and Private health services	168	35.6%	125	
**Use of medication**				
Frequent use of medication	115	24.4%	124	W = 19545 (0.4398)
Do not uses medication frequently	357	75.6%	125	
**Previously diagnosed health condition**				
No	346	73.3%	125	KW =32.668 (< 0.001)
Diabetes	17	3.6%	118	
Cardiovascular diseases	6	1.3%	109	
Hypertension	45	9.5%	121	
Depression	54	11.4%	131	
Anxiety	9	1.9%	133	
Cancer	4	0.8%	124	
Other	24	5.1%	120	

*KW* stands for Kruskal-Wallis test; *D* for Dunn’s test; *W* for Wilcoxon test

### Mental health literacy scale

The MHLS encompasses 35 items, and comprises six attributes: (a) ability to recognize specific disorders; (b) knowledge of how to seek mental health information; (c) knowledge of risk factors and causes; (d) knowledge of self-treatments; (e) knowledge of available professional help; and (f) attitudes that promote recognition and appropriate help-seeking [11]. Items related to attributes "a" and "c" contain response options on a four-point scale ranging from very unlikely (1) to very likely (4). Items related to the attribute "d" also have answers on a four-point scale, but vary between very useful (4) and very useless (1). Items related to attributes "b", "e", and "f" contain response options that vary on a five-point scale, from completely agree (5) to completely disagree (1). MHLS scores are calculated by summing the scores for each item, ranging between 35 and 160 points, so that the higher the score is, the greater the mental health literacy.

The MHLS has been widely used and validated in several languages to measure mental health literacy [17, 19, 22, 23]. This scale was translated into Brazilian Portuguese by [25], who performed forward and backward translations, in addition to semantic validation practitioners psychologists and nurses. However, MHLS has not yet been validated in this language.

### Analysis

Descriptive statistics were used to present the sociodemographic profile of the sample, in addition to the MHLS scores. Wilcoxon tests were applied to compare the statistical significance of the differences between the medians of the MHLS scores of independent groups. These groups were formed based on different socioeconomic characteristics. Kruskal-Wallis tests were used for samples with more than two independent groups. In situations where statistical significance was observed, Dunn’s post-hoc test was performed. These methods are appropriate for ordinal or quantitative data and do not depend on the assumption of normal distribution [26].

The authors conducted factor analyses to examine the hypothesis of unidimensionality and to identify a more appropriately structured model for the MHLS. Before running the factor analyses, the instrument’s internal reliability was verified under the assumption of unidimensionality. Cronbach’s $\alpha$ and McDonald’s $\omega$ tests were employed to evaluate internal consistency. In addition, the Kaiser-Meyer-Olkin (KMO) test was conducted to verify whether the sample was appropriate for discriminatory analyses. This test measures the relationship between the total and partial correlations of the variables, and its result is a measurement of the sample adequacy (MSA), which varies between 0 and 1. Values closer to 1 indicate sample adequacy for the analyses. The Bartlett sphericity test was performed to check whether the correlations between the variables were different from those in identity matrix. A significant result indicates that there are significant relationships between the variables.

A confirmatory factor analysis was conducted using the free software R to test the hypothesis of unidimensionality. This special type of structural equation modeling involves multiple regression analyses of factors to test the relationships between observed and latent variables [27].

Exploratory factor analyses (EFA) were then carried out with the Sample to test the structure of a better adjusted model for the MHLS. This technique uses the variance shared by the observed variables to group them into factors. It is noteworthy that the sample size (472) is more than sufficient to carry out EFA, since the ideal N for extracting factors with factor loadings of 0.4 would be 300 [28]. We used the principal axis factoring estimation method and oblimin rotation, as carried out by the authors of the MHLS O’Connor and Casey (2015).

To extract the factors, the latent values of the factor loadings were ordered in a graph (screeplot), and their inflection point was observed. Furthermore, the sizes of the eigenvalues were also observed, restricting themselves to vectors with eigenvalues greater than 1 [27]. The number of factors was restricted to those that had at least three salient variables [28].

The internal consistency reliability of the identified structure was verified in three stages: exploring responses to the items; adjusting the measurement model to the data, which included a confirmatory factor analysis; and calculating the reliability coefficient derived from the model’s parameters. The last step covered not only the calculation of McDonald’s Omega but also the verification of the behavior of this indicator when each item in the measurement model is excluded — referred to as ‘Omega without the item’ [29].

Finally, we evaluated the invariance of the psychometric instrument across different demographic groups to determine if the scale’s performance varies. Specifically, we divided the sample by gender and assessed three types of invariance: configural invariance, to verify whether the proposed structure is consistent across these groups; metric invariance, to ensure that factor loadings are equivalent across the groups; scalar invariance, to check if the intercepts of the items remain consistent across the different groups [30].

## Results

Although not the objective of the research, the significant differences found between the level of mental health literacy for different groups of respondents based on sociodemographic characteristics deserve to be reported. Women had significantly higher MHL levels than men did (W = 32728, *p*-value < 0.001). Significant differences were also observed in the level of literacy depending on age (KW = 39.015, *p*-value < 0.001), with higher levels of literacy being observed among the youngest (18-25 and 26-30 years old) and oldest (over 50 years old) participants.

Education is another characteristic in which significantly different levels of MHL are observed (KW = 45.958, *p*-value < 0.001). People with higher levels of education are those who have higher literacy scores. Notably, the difference in literacy between people with incomplete higher education and those with complete higher education was significantly greater (D = −2.297, *p*-value = 0.010).

People who exclusively used private health services had significantly greater health literacy than did those who exclusively used public health services (D = 2.846, *p*-value = 0.002). Significant differences in the level of literacy are also observed depending on previously diagnosed health conditions (KW = 32.668, *p*-value < 0.001). People with common mental disorders, such as depression and anxiety, have significantly greater literacy, and people with diabetes and cardiovascular diseases have significantly lower health literacy.

Finally, no significant differences were observed depending on race (KW = 8.283, *p*-value = 0.08172), labor situation (KW = 0.010, *p*-value = 0.9947), income (KW = 7.801, *p*-value = 0.167), housing condition (W = 15098, *p*-value = 0.354), or use of medication (W = 19545, *p*-value = 0.4398).

### Confirmatory factor analysis

The single-factor structure, as proposed by O’Connor and Casey (2015), was tested through CFA. The unidimensional model presented poor fit indices. This can be observed through the comparative fit index (CFI = 0.558) and the Tucker-Lewis index (TLI = 0.531). The closer to one, the better adjusted the model is [31]. The root mean square error of approximation (RMSEA = 0.077) and standardized root mean square residual (SRMR = 0.081) address model errors or residuals. The lower the RMSEA and SRMR are, the better the model fit [31]. Therefore, they also indicate that the models have poor fit. In sum, the single-factor structure is not recommended.

### Exploratory factor analysis

The Cronbach’s $\alpha$ value for this sample is 0.83, and the McDonald’s $\omega$ is 0.86, indicating reliability. The KMO test presented an overall value of 0.85, indicating that the sample has a good fit. However two items did not show good adjustment, x10 (msa = 0.5) and x18 (msa = 0.52). The Bartlett test presented a highly significant result (chi-square = 3185.366, *p*-value < 0.001), thus confirming the adequacy of the sample for factor analysis.

Screeplot analysis indicates a five-factor model. Accordingly, only five factors had eigenvalues greater than 1. However, this model is statistically underidentifiable, as the fifth factor presents only one item. Therefore, a four-factor structure is the most viable. The cutoff point applied to define the factors was 0.4, that is, variables with factor loadings higher than this cutoff point were selected to compose the factors. The factor loadings for the items vary between 0.441 and 0.769, which indicates good quality [32]. The alternative fit indices TLI = 0.932, RMSEA = 0.025 and SRMR = 0.04 indicate a good fit of the model [31].

The proportion of variance explained by the model is 58%. The correlations between the factors range from 0.09 to 0.42, that is, they are moderate to low, indicating that the factors are relatively distinct, but there is some overlap between the factors related to behavior. Multicollinearity problems between the items were not identified, indicating that there was no redundancy between them.

Table 2 presents the structure of the four-factor model estimated in EFA, the factor loadings and uniquenesses of the items. This is a shorter version of the MHLS model proposed by O’Connor and Casey (2015), containing 18 items and four factors, which cover aspects related to: social stigma behavior, behavior of denying the disorder, skills to recognize disorder and seek treatment, and knowledge about where to look for information.

**Table 2:** Structure of the MHLS short version

Item	Factor Loading	Uniqueness
*Factor 1 – Attitudes toward people (Social Stigma Behavior)*		
x29	0.505	0.748
x30	0.640	0.562
x31	0.720	0.466
x32	0.711	0.515
x33	0.639	0.591
x34	0.592	0.597
x35	0.755	0.437
*Factor 2 - General attitudes toward MH (Behavior of Denying the Disorder)*		
x20	0.441	0.719
x21	0.664	0.515
x24	0.556	0.611
x26	0.642	0.601
x27	0.454	0.788
*Factor 3 - MH Recognition (Skills to Recognize Disorder and Seek Treatment)*		
x1	0.458	0.791
x3	0.447	0.780
x6	0.444	0.785
*Factor 4 - Information Seeking (Knowledge about Where to Look for Information)*		
x16	0.523	0.699
x17	0.475	0.763
x19	0.769	0.415

To determine if the reduced scale measures the same construct as the original scale, we calculated the correlation between the total scores of each observation from both scales. The analysis yielded a correlation coefficient of 0.948 (*p*-value < 0.001), demonstrating a strong positive correlation. This finding indicates that the reduced scale maintains consistency with the original scale.

### Internal consistency reliability

Initially, the variables used in the model consist of either five or four ordered categories, representing ordinal data obtained through a Likert-type scale. A possible ceiling effect was observed in most variables, as can be seen in Table 3. Additionally, this study includes a large sample, totaling 472 respondents. Thus, the WLSMV estimator tends to be the most suitable for estimating the structural equation model [29].

**Table 3:** Descriptive statistics of variables

Variable	% 1	% 2	% 3	% 4	% 5
x29	7.42	22.25	19.92	33.26	17.16
x30	17.58	21.19	8.47	35.59	17.16
x31	3.60	5.30	5.51	47.88	37.71
x32	4.66	12.29	11.02	41.53	30.51
x33	7.84	13.35	15.89	27.54	35.38
x34	21.61	15.04	9.11	35.81	18.43
x35	5.72	13.56	10.17	51.91	18.64
x20	6.78	14.83	5.08	31.57	41.74
x21	4.66	11.65	2.12	17.37	64.19
x24	2.75	8.47	2.54	23.09	63.14
x26	3.81	4.87	2.33	12.29	76.69
x27	3.18	4.03	1.91	10.81	80.08
x1	3.81	19.28	66.10	10.81	0.00
x3	5.30	17.58	56.99	20.13	0.00
x6	4.87	20.55	64.19	10.38	0.00
x16	5.72	18.01	6.14	32.20	37.92
x17	6.78	16.31	5.72	36.65	34.53
x19	5.72	8.69	4.24	31.36	50.00

We then evaluated the short version model’s fit to the data using confirmatory factor analysis. The results indicate a well-fitted model, as evidenced by the null $\chi ^2$ value and alternative fit indices (CFI = 0.991, TLI = 0.989, RMSEA = 0.04, SRMR = 0.057), providing favorable evidence of the measurement model’s validity.

Finally, we calculated the McDonald’s $\omega$ of the model, as well as the ‘omega without the item’. The results indicate the reliability of the internal consistency: the total $\omega$ was 0.85, and the ’omega without the item’ values ranged from 0.83 to 0.85, indicating that removing any of the items would not increase the internal consistency of the model.

### Invariance assessment

Invariance tests were conducted to determine if the structure and parameters of the Short Mental Health Literacy Scale are consistent across different groups, enabling valid comparisons of scores between these groups [30]. Configural invariance was assessed without imposing restrictions across groups, and the results confirmed that the proposed factor structure is preserved across the different groups evaluated. Metric invariance was tested by ensuring that factor loadings are equivalent across groups. The model fit did not significantly deteriorate, indicating that the factor loadings are uniform. Scalar invariance was assessed by equalizing the intercepts of the items across groups, and the findings suggest that the factor means are comparable between the groups. Table 4 shows the results of the invariance tests considering different gender and education groups.

**Table 4:** Factorial invariance tests

Model	DF	AIC	BIC	$\varvec{\chi }^{\varvec{2}}$	$\varvec{\Delta \chi }^{\varvec{2}}$	$\varvec{\Delta }$ DF	*p*-value
Configural	258	23342	23841	521.98	-	-	-
Metric	272	23336	23776	543.14	21.159	14	0.0976
Scalar	286	23327	23709	562.19	19.047	14	0.1632

## Discussion

The research findings revealed that the Brazilian Portuguese version of the MHLS exhibited a four-factor structure, in a shorter version, containing 18 items with adequate factor loadings and high reliability indices. These aspects indicate that the instrument is internally consistent and reliable, and is effective in measuring mental health literacy.

In agreement with what was found in other attempts to validate the MHLS, the confirmatory factor analysis indicated that the model with a single-factor structure does not present a good fit. In fact, the four-factor structure, which was found to be the most appropriate for the MHLS, is consistent with the findings of other studies [17, 22, 23]. The validation of the instrument for Portugal [16] revealed a set of items similar to that found in this study but with a different structure. The European Portuguese version has three factors.

Although the structure is similar, the present version is shorter than the Slovenian, Saudi Arabian, and Vietnamese versions, considering that the factor loadings of some items were lower. This is probably because the sample in this research is more heterogeneous than the others. This can be seen by the greater flattening of the distribution of MHLS values in the sample of the present study (Kurtosis: 0.684) in relation to the others O’Connor and Casey (2015) = −0.231; Krohne et al. (2022) = 0.41; BinDhim et al. (2023) = −0.05, suggesting that there is more variation in the scores, with a greater number of individuals with very high or very low results than in the other versions of the MHLS.

The differences in scales validated for different contexts can be attributed to variations in population characteristics and sampling methods. This study utilized a representative sample from the Federal District in Brazil, a region distinguished by its diverse and mixed population due to historical migration from various parts of Brazil. Consequently, this area displays greater diversity and socioeconomic disparity compared to countries like Saudi Arabia [17] and Slovenia [23], featuring significant ethnic, social, economic, and cultural distinctions. In contrast, other studies, such as the one by O’Connor and Casey (2015), used sampling methods like the snowball technique, which tends to produce more homogeneous samples by involving participants who are interconnected.

Additionally, the construct of mental health literacy may evolve over time, suggesting that future studies could explore the development of new scales to better capture this dynamism. Nevertheless, our research provides critical insights into the validity of the MHLS within a heterogeneous and culturally diverse setting in the global south. It lays a solid foundation for subsequent studies aimed at evaluating the construct’s validity across various Brazilian demographics, extending work previously initiated in Buta et al. [24].

However, regarding the distribution of scores, the scores in the present study were more similar to those found by the Slovenian and Saudi Arabic versions [17, 23] than to those of the original Scale [11], which is quite asymmetric. The asymmetry to the left suggests that more people tend to have higher mental health literacy scores, with fewer individuals presenting very low scores.

Categories adopted for the factors were similar to those adopted in the Slovenian and Saudi Arabic versions[17, 23], covering: attitudes towards people with mental disorders, which refers to aspects related to social stigma behavior; general attitudes toward mental health, related to the behavior of denying the disorder; mental health recognition, referring to the skills to recognize disorder and seek treatment; and information seeking, related to the knowledge about where to look for information.

The results demonstrated a strong positive correlation between the total scores from both scales, indicating that the reduction in the number of items did not affect the construct. Additionally, the theoretical aspect was considered, revealing substantial similarities with the literature, as previously noted. It is important to mention that the remaining items encompass the attributes stated in the original scale, except for those that include specific aspects of Australian culture (e.g., ‘Knowledge of risk factors and causes’) or those requiring very specific or in-depth technical knowledge (e.g., ‘To what extent do you think it is likely that Cognitive Behavior Therapy (CBT) is a therapy based on challenging negative thoughts and increasing helpful behaviors’, or ‘To what extent do you think it is likely that Dysthymia is a disorder’). In short, adapting the MHLS to Portuguese offers a more parsimonious scale, facilitating its application with potential research subjects.

The average score of the present sample is 123.01, which is lower than that found by O’Connor and Casey (2015), 127.38 for Australian adults; Brooker and Tocque (2023), 128 for probation staff in European countries; and Dias et al. (2021), 129.1 for Portuguese adults. However, several studies reported lower overall MHLS scores; for example, Dang et al.(2018) reported a score of 106.15 for Vietnamese teachers; Dessauvagie et al. (2022) reported a score of 108.13 for Vietnamese and Cambodian university students; Krohne et al. (2022) reported a score of 114.09 for the Slovenian general population; BinDhim et al. (2023) reported a score of 115.5 for Arabic-speaking Saudi adults; and Snow et al. (2023) reported a score of 121 for Polynesian adults residing in the USA.

We observed a difference in mental health literacy according to gender, with women having higher MHL than men. This finding is in line with most of the literature [14–20]. These findings may reflect strong cultural characteristics. Men tend to perceive the recognition of mental disorders, the expression of emotional challenges, and the seeking of external help as signs of personal weakness and typically feminine behaviors [15, 33].

Age also proved to be a relevant factor for the MHLS score. We observe higher levels of literacy among young adults (18–30 years old) and people over 50 years old. A greater prevalence of mental health literacy was observed in individuals aged 30 to 40 years in some studies thatincluded general samples of the community [15, 16, 19]. Others observe higher levels of mental health literacy among younger groups (18-22) [14, 20] These studies, however, focus on university students. There are still those who do not observe significant differences in the level of MHLS between different age groups[18]. However, it has been observed in the literature that people over fifty years of age have lower levels of health literacy [15, 18], which could be related to a negative attitude toward mental health, given the loss of vocational activity related to retirement [15], or even intergenerational cultural differences.

We also observed higher MHL scores in people with higher educational levels. This finding is in line with the literature [13, 14, 16–20]. However, it is interesting to note that groups with ongoing undergraduate courses had higher MHL scores than groups of people who had completed higher education.

The type of health service used by the respondents was another factor related to the level of MHL. Those who use private services have greater literacy than those who use public services. Although no significant differences were observed depending on income, this result may be related to income, since people who exclusively use private health services tend to have higher income levels than those who use public services. In fact, not having observed significant differences in MHS depending on income may be a bias related to this sample, since this is a factor described in the literature as capable of influencing MHL [19, 20].

Furthermore, there is evidence of significant differences in MHL between groups of people with certain health conditions. People who suffer from common mental health disorders, such as depression and anxiety, tend to have higher MHLS scores. This finding is consistent with other studies reporting higher levels of literacy in people previously diagnosed with mental disorders [11, 14, 17, 18, 20]. It is possible that people who have been diagnosed and accepted such a diagnosis are more likely to seek help. In fact, people who are aware of mental health issues tend to be more inclined to seek professional help [20]. In addition, prior contact with mental health professionals tends to increase knowledge about personal treatments.

Finally, no significant differences in MHL were observed depending on race, labor situation, housing condition, or use of medication. There are indications in the literature about the relationships between MHL and labor situation and housing conditions, which contradicts these findings. People who lose their vocational activity tend to have negative mental health outcomes. In addition, married people who have greater family support tend to experience positive attitudes toward mental health [15].

This study has some limitations. First, it is important to state that the sample was not obtained randomly, so the results cannot be extrapolated to the population. Also, the study is based on a translation of the MHLS previously made by other researchers, so the authors had no control over this process. Moreover, other factors not assessed here may be related to mental health literacy, such as profession, history of mental disorders in family members, educational level of the respondents’ parents, etc. This should be observed in future studies. In addition, the differences found in the MHL across various sociodemographic characteristics and health conditions are based on direct peer comparisons within these specific groups. To ensure that these differences are not affected by other variables, other analyses are needed.

Despite these limitations, it should be highlighted that no previous MHLS evaluations carried out with Brazilians were found. Therefore, to the best of our knowledge, this is the first study that measures the MHLS in a sample of Brazilians. In addition, a shorter version of the MHLS adheres to the principle of parsimony, allowing a few items to measure the same construct effectively. This simplification enhances the instrument’s applicability and the efficiency of the data collection process. Furthermore, the four-dimensional structure provides significant informational gains by enabling the distinction of various aspects that constitute the MHL construct, which a one-dimensional structure cannot achieve. This distinction allows to identify which aspects of literacy individuals need greater support in.

## Conclusion

This study aimed to validate a version of the MHLS adapted to Brazilian Portuguese. Unlike the original 35-item unidimensional scale, the results of this study pointed to an 18-item scale composed of four factors, which exhibited reliable internal consistency. The Brazilian version of the MHLS has proven to be a valuable instrument for measuring the mental health literacy of Brazilians. In any case, slight variations in wording can considerably influence the interpretation of statements in different cultural contexts. Thus, additional validations should be carried out.

Furthermore, this study revealed that levels of mental health literacy depend on the sociodemographic characteristics of the sample. Identifying groups most likely to hold negative or mistaken views about mental health is an initial step toward developing strategic targeted interventions. The evidence demonstrates the need for strategies to expand mental health literacy and reduce the social stigma surrounding mental disorders to target middle-aged, under-educated men who use public services. However, further studies are needed to understand these relationships in other populations.

## Data Availability

The data will be made available to interested parties upon reasonable request.
